# Percutaneous laser ablation of benign thyroid nodules: a one year follow-up study

**DOI:** 10.31744/einstein_journal/2018AO4279

**Published:** 2018-11-29

**Authors:** Antonio Rahal, Priscila Mina Falsarella, Guilherme Falleiros Mendes, Jairo Tabacow Hidal, Danielle Macellaro Andreoni, José Flávio Ferreira Lúcio, Marcos Roberto Gomes de Queiroz, Rodrigo Gobbo Garcia

**Affiliations:** 1 Hospital Israelita Albert Einstein, São Paulo, SP, Brazil.; 2 Escola Paulista de Medicina, Universidade Federal de São Paulo, São Paulo, SP, Brazil.

**Keywords:** Lasers, Ablation technique, Thyroid nodule, Brazil, Lasers, Técnicas de ablação, Nódulo da glândula tireoide, Brasil

## Abstract

**Objective:**

To evaluate safety and effectiveness of nodule volume reduction and thyroid function after percutaneous laser ablation treatment in patients with benign nonfunctioning thyroid nodules.

**Methods:**

Prospective single-center study, from January 2011 to October 2012, which evaluated 30 euthyroid and thyroid antibodies negative patients with benign solitary or dominant nodule with indication of treatment due to compressive symptoms and aesthetic disturbances. The clinical and laboratory (thyroid ultrasound, TSH, FT4, TG, TG-Ab, TPO-Ab and TRAb levels) evaluations were performed before the procedure, and periodically 1 week, 3 months and 6 months after. The ablation technique was performed under local anesthesia and sedation. In each treatment, one to three 21G spinal needle were inserted into the thyroid nodule. The laser fiber was positioned through the needle, which was then withdrawn 10mm to leave the tip in direct contact with the nodule tissue. Patients were treated with a ND: Yag-laser output power of 4W and 1,500 to 2,000J per fiber per treatment. The entire procedure was performed under US guidance.

**Results:**

Thirty patients, with a total of 31 nodules submitted to laser ablation were evaluated. The median volumetric reduction of the nodule was approximately 60% after 12 months. No statistical significance was observed on thyroid function and antibodies levels. There was a peak on the level of thyroglobulin after the procedure due to tissue destruction (p<0.0001). No adverse effects were observed.

**Conclusion:**

Percutaneous laser ablation is a promising outpatient minimally invasive treatment of benign thyroid nodule.

## RESUMO

**Objetivo:**

Avaliar a segurança e a efetividade da redução de volume nodular e função tireoidiana após tratamento com ablação percutânea por laser em pacientes com nódulos tireoidianos benignos não funcionantes.

**Métodos:**

Estudo unicêntrico prospectivo, de janeiro de 2011 a outubro de 2012, que avaliou 30 pacientes eutireoideos (com anticorpos antitireoide negativos), com nódulo solitário ou dominante benigno, com indicação de tratamento devido a sintomas de compressão e distúrbios estéticos. As avaliações clínica e laboratorial (ultrassonografia de tireoide, TSH, FT4, TG, TG-Ab, TPO-Ab e TRAb) foram realizada antes do procedimento e periodicamente − 1 semana, 3 meses e 6 meses depois. A técnica de ablação consistiu em procedimento realizado sob a anestesia local e sedação. Em cada tratamento, uma a três agulhas espinhais 21G foram inseridas no nódulo tireoidiano. A fibra laser foi posicionada através da agulha que foi, então, retirada 10mm, para deixar a ponta em contato direto com o nódulo. Os pacientes foram tratados com uma potência de saída ND: Yag-laser de 4W e 1.500 a 2.000J por fibra por tratamento. Todo o procedimento foi guiado por ultrassonografia.

**Resultados:**

Foram avaliados 30 pacientes, com total de 31 nódulos submetidos à ablação a laser. A redução média volumétrica do nódulo foi de aproximadamente 60% após 12 meses. Não foi observada significância estatística na função da tireoide e nem nos níveis de anticorpos. Houve pico no nível de tiroglobulina após o procedimento devido à destruição do tecido (p<0,0001). Nenhum efeito adverso foi observado.

**Conclusão:**

A ablação com laser é um tratamento minimamente invasivo promissor para tratamento do nódulo benigno da tireoide.

## INTRODUCTION

Long-term management of benign thyroid nodules is a problem in clinical practice. The majority of nodules are clinically followed up with ultrasonography (US). However, some nodules will grow and present aesthetic disturbances or local compressive symptoms (neck pain, dysphagia and cough).^(^ ^1^ ^)^ Currently, the standard treatment is surgical, however the risks of associated short- and medium-term complications are cause for concern.^(^ ^2^ ^)^

Over the last two decades, several minimally invasive modalities guided by image have been proposed for the treatment of benign thyroid nodules.^(^ ^1^ ^)^ Ultrasound (US)-guided percutaneous ethanol injection is recognized as an effective and inexpensive treatment for thyroid cystic lesions,^(^ ^3^ ^)^ and laser thermal ablation is usually employed in solid thyroid nodules.^(^ ^1^ ^)^

Two randomized trials showed that US-guided percutaneous laser ablation (PLA) is a safe and effective therapeutic option as an alternative to surgery for benign symptomatic nodules, with an average decrease in nodule volume of over 40 to 50% after 1 year.^(^ ^4^ ^,^ ^5^ ^)^ Many other uncontrolled studies showed that PLA is well tolerated with complication rate less than 3%. Most common side effects were transient pain, fever and mild skin burn.^(^ ^6^ ^,^ ^7^ ^)^ Transient dysphonia (1 week to 2 months) due to vocal fold paresis was described in very few patients,^(^ ^4^ ^,^ ^5^ ^)^ and one case of tracheal laceration was reported.^(^ ^8^ ^)^

The incidence of hypothyroidism after hemithyroidectomy for benign nodule varies from 15 to 49%.^(^ ^9^ ^,^ ^10^ ^)^ Valcavi et al., in a large series of patients treated by PLA, demonstrated that the incidence of hypothyroidism after treatment was only 1.6%,^(^ ^11^ ^)^ thus avoiding long-term levothyroxine supplementation in many patients.

## OBJECTIVE

To evaluate safety and efficacy of nodule volume reduction and thyroid function after percutaneous laser ablation treatment in Brazilian patients, with benign nonfunctioning thyroid nodules, in 1-year follow-up.

## METHODS

### Patients

Thirty patients aged 18 to 84 years, with benign solitary or dominant nodule, were recruited from the Thyroid Disease Clinic at the hospital, between January 2011 and October 2012. Fine-needle aspiration biopsy was performed confirming colloid nodule, and was repeated after 4 months, by the same cytologist, to confirm the result. All patients had indication of treatment due to compressive symptoms, aesthetic disturbances, large size, contraindication for surgery, or refusal of the patient to undergo surgery (for aesthetic concerns). None had a family history of thyroid carcinoma or were submitted to neck radiation. All patients were euthyroid with negative thyroid antibodies and normal calcitonin levels prior to the procedure (Table 1). Patients with coagulation problems were excluded.

**Table 1:** Patients’ characteristics and mean laboratory tests prior to percutaneous laser ablation procedure

Characteristics	Baseline values
Number of patients	30
Age, years	18-84
Age, years	46.06
Sex	
Female	29
Male	1
Nodule volume, mL	12.44 (1.4-61.4)
TSH, mcIU/mL	1.31
FT4, ng/dL	1.15
Tg, ng/mL	122.51
TgAb, IU/mL	15.66
TPOAb, IU/mL	15.00
TRAb, UI/L	0.46

TSH: thyroid-stimulating hormone; FT4: serum-free thyroxine; Tg: thyroglobulin; TgAb: antithyroglobulin; TPOAb: antithyroid peroxidase; TRAb: anti-TSH-receptor antibodies.

The Institutional Review Board (number 10/1469) of our hospital approved this protocol, and all the patients signed Informed Consent before the treatment.

### Follow-up evaluation

The clinical and laboratory evaluation was performed before the procedure and periodically, after 1 week, 3, 6 and 12 months. It consisted of physical examination, thyroid US, thyroid-stimulating hormone (TSH), serum-free thyroxine (FT4), thyroglobulin (Tg), antithyroglobulin antibodies (antiTgAb), antithyroid peroxidase antibodies (anti-TPOAb) and anti-TSH-receptor antibodies (TRAb) dosages. Calcitonin was measured prior PLA, and 1 year after the procedure.

Thyroid US was performed with Esaote system MyLab™ 70XVG, 12MHz linear transducer (Esaote, Genoa, Italy), the same used during the procedure. The nodule volumes were calculated by the ellipsoid formula, multiplying by 0.52 the three diameters in cm (width, length and depth).

The blood test reference values were TSH: 0.4 to 4.5mcUI/mL (chemiluminescence assay third generation kit); FT4: 0.78 to 2.19ng/dL (chemiluminescence assay); Tg: 6 to 50ng/mL (chemiluminescent immunometric assay); antiTgAb: 0 to 115IU/mL (chemiluminescent immunometric assay); anti-TPOAb: 0 to 34IU/mL (electrochemiluminescent assay); TRAb: <1.22UI/L (electrochemiluminescent assay); and calcitonin; 5 to 11pg/mL (chemiluminescent immunometric assay).

### Ablation technique

**Figure 1:**
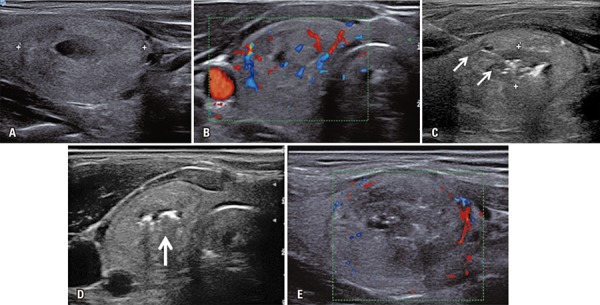
Ultrasonography of thyroid after procedure showing absence of central vascularization. (A) Pre-ablation ultrasonography with a 3cm on the largest axis predominantly solid thyroid nodule in the right lobe. (B) Insertion of the optic fiber (arrows) into the thyroid nodule. (C) Gas formation (arrow) during the procedure. (D) Ultrasonography of thyroid after procedure showing increased hypoechogenicity and absence of central vascularization. (E) Ultrasonography of thyroid after procedure showing absence of central vascularization

The procedure was performed in the interventional medicine center of the organization. All patients had fasted for 8 hours. A conscious sedation with 30 to 50mcg of fentanyl citrate intravenous injection was given to all patients. Local anesthesia with 2% lidocaine was injected in the subcutaneous tissue and subcapsular region of the thyroid gland. In each treatment, one to three (depending on nodule volume) 21G spinal needle were inserted into thyroid nodule by US guidance. The needle entry point was preferably the isthmus of thyroid, however the access used varied according to the location of the nodule. For nodules with a volume smaller than 15mL, one or two needles were used, and for those greater than 15mL, three needles. The laser fiber was positioned through the needle and later withdrawn 10mm to leave the tip in direct contact with the nodule tissue.^(^ ^12^ ^)^ Patients were treated with a ND: Yag laser output power of 4W, and 1,500-2,000J per fiber per treatment. In some cases needle pull-backs were performed and additional energy was administered. Each treatment lasted 5 to 10 minutes. The entire procedure was performed under US-guidance (Figure 1 A-1E). After the procedure all patients received a betamethasone 5mg intramuscular injection, and waited for 2 hour in recovery room prior to hospital discharge.

### Statistical analysis

Results are given as mean, standard deviation and range. Friedman’s test and Wilcoxon were used to compare data within and between groups. A p value <0,05 was considered significant. The statistical analyses were performed using the Statistical Package of the Social Science statistical software, version 20.0 (SPSS, Chicago, Illinois, USA).

## RESULTS

**Figure 2:**
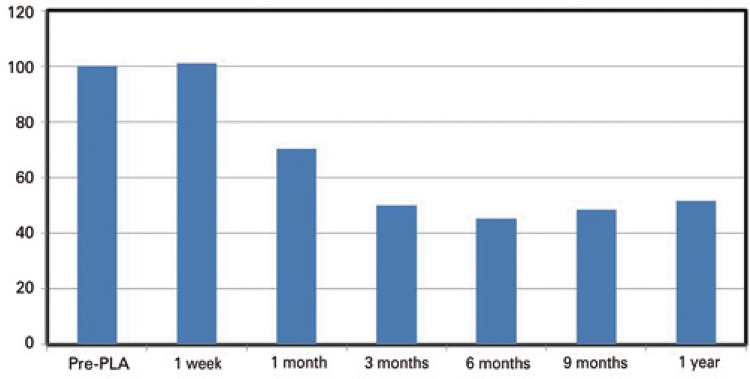
Percentage reduction of nodules

A total of 30 nodules were examined. The nodule largest diameter ranged from 14mm to 68mm (median 29.5mm), and the median nodule volume was 12.44cm^3^, ranging from 1.4 to 61.4cm^3^. All, but one nodule, responded to the treatment. One week after the procedure, the nodules got slightly bigger because of the edema caused by ablation. One month later, the volume shrinkage could already be seen. The average reduction 1 year after the procedure was 53% (Figure 2 and Table 2).

PLA: percutaneous laser ablation.

**Table 2:** Mean nodule volume and blood tests during the 1-year follow-up

Time after PLA	Pre-treatment	1 week	1 month	3 months	6 months	9 months	1 year
Nodule volume, mL	12.44±15.10	11.74±13.55	9.15±12.76	6.66±9.80	6.78±10.61	7.72±10.83	6.81±10.23
TSH, mcIU/mL	1.31±0.95	1.17±0.87	1.38±1.00	1.69±1.44	1.70±1.29	2.21±1.89	1.70±1.19
FT4, ng/dL	1.15±0.16	1.31±0.33	1.14±0.20	1.12±0.17	1.10±0.13	1.20±0.17	1.10±0.12
Tg, ng/mL	12.25±126.26	96.54±1389.37	11.66±128.76	12.33±148.80	10.20±108.28	8.40±120.89	14.64±155.46
TgAb, IU/mL	15.20±٧.84	19.92±15.13	18.94±15.93	32.81±80.10	20.77±20.34	15.37±7.88	22.07±25.87
TPOAb, IU/mL	24.48±54.05	22.02±49.41	18.00±31.96	32.772±85.08	23.18±45.93	18.5±40.34	14.63±19.75
TRAb, IU/L	0.46±٠.25	0.62±0.29	0.62±0.34	0.68±0.35	0.73±0.31	0.57±0.27	0.63±0.22

Results expressed as mean±standard deviation. TSH: thyroid-stimulating hormone; FT4: serum-free thyroxine; Tg: thyroglobulin; TgAb: antithyroglobulin; TPOAb: antithyroid peroxidase; TRAb: anti-TSH-receptor antibodies.

**Figure 3:**
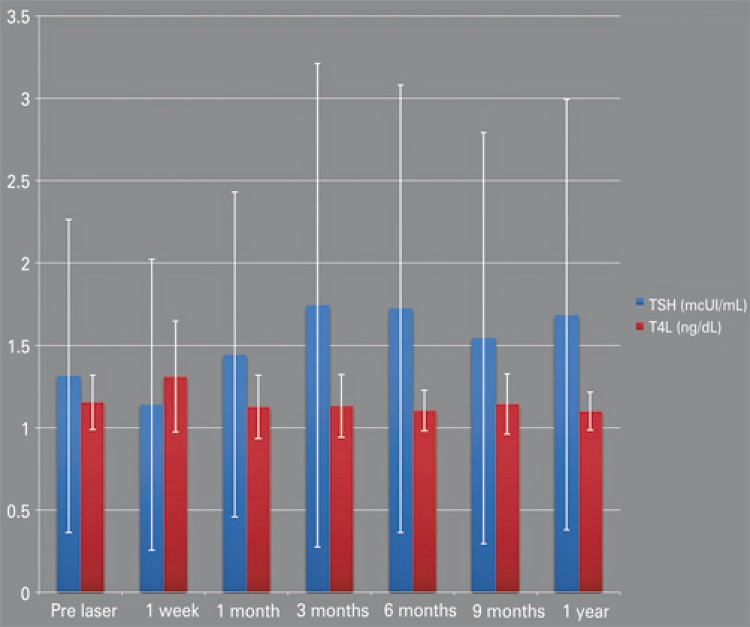
Thyroid-stimulating hormone and serum-free thyroxine mean levels

TSH: Thyroid-stimulating hormone; FT4: serum-free thyroxine.

Serum concentrations of TSH and FT4 were normal in all cases and did not change during the follow-up (p=0.440 and p=0.565, respectively) (Figure 3).

**Figure 4:**
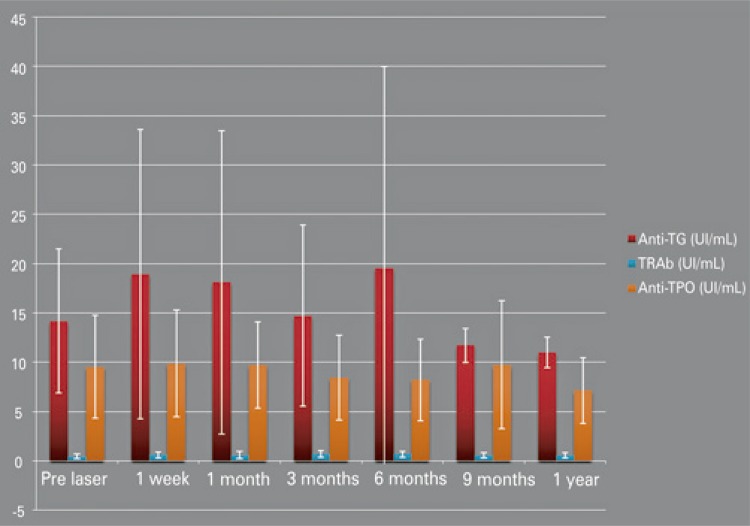
Anti-thyroid-stimulating hormone-receptor antibodies, antithyroglobulin, antithyroid peroxidase mean levels

TRAb: Anti-thyroid-stimulating hormone-receptor antibodies; anti-Tg: antithyroglobulin; anti-TPO: antithyroid peroxidase.

All patients had negative antibodies (TgAb and TPOAb) before the laser ablation. After the procedure, they remained negative (p=0.250 and p=0.083, respectively). Serum calcitonin was also negative before and after treatment. TRAb became positive in three patients during the follow-up but, after 1 year, they were all negative again (p=0.295) (Figure 4).

**Figure 5:**
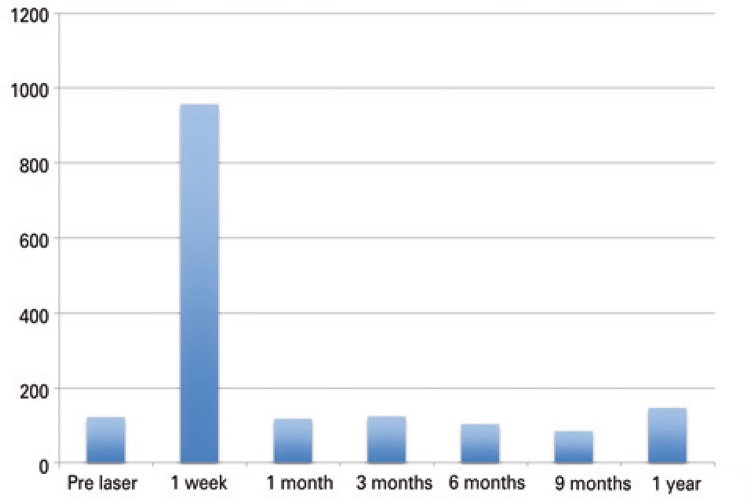
Evolution of thyroglobulin

Thyroglobulin levels presented an important increase soon after the procedure (Fridman p<0.01), followed by a significant reduction 1 month after (Figure 5). The Tg levels returned to pre-treatment levels after 1 year.

Only one patient reported pain on the site of needle insertion after treatment but it resolved with ibuprofen for 3 days.

Considering the complications of the procedure, only 3 out of 30 patients (10%) reported mild pain, but it was alleviated with ibuprofen for 3 days. One patient had a burn lesion on the skin where the needle was inserted, that recovered within 10 days, without leaving a scar.

All patients also filled in a questionnaire about satisfaction with treatment that considered intensity of symptoms before and after the procedure, and general satisfaction. Twenty-nine patients were totally satisfied, while one (3%) reported a slight improvement in symptoms − this subject was the only one that did not respond to treatment.

## DISCUSSION

Percutaneous laser ablation is an alternative to surgery in the management of benign thyroid nodule with compressive symptoms or aesthetic disturbances. Since its first description by Pacella et al.,^(^ ^6^ ^)^ numerous studies provided consistent evidence of clinical efficacy of laser ablation in benign thyroid nodules.^(^ ^12^ ^,^ ^13^ ^)^ Papini et al.,^(^ ^1^ ^)^ in a randomized study of 200 patients, comparing laser ablation and clinical observation in patients with benign thyroid nodules, demonstrated a significant and persistent reduction in the volume of the laser-treated nodules associated with improvement of the associated local symptoms, with no change in thyroid function.

Some authors demonstrated that reduction of the nodule is proportional to the energy administered.^(^ ^5^ ^,^ ^13^ ^)^ In our center, we also observed that higher energy levels led to higher nodule shrinkage. In order to achieve a bigger ablated area on the nodule the needle pull-back technique described by Valcavi et al.,^(^ ^11^ ^)^ was used. The US performed after the procedure showed an avascular central area that corresponded to the ablated area.

Likewise the prior studies, an increased nodule volume was observed in the first week after PLA, probably due to thyroid tissue edema.^(^ ^11^ ^)^ Literature shows that the percentage of nodule reduction does not depend on its initial size.^(^ ^4^ ^,^ ^6^ ^,^ ^11^ ^,^ ^13^ ^)^ Our data, on the other hand, demonstrated better results in nodules smaller than 4mL. In this group the reduction rate was approximately 83%.

Only euthyroid patients, who are negative for thyroid antibodies, were selected to assess the impact of treatment in the thyroid function. Since PLA expose thyroid antigen, it was a concern to check development of thyroid autoimmunity. In our data, no cases of antibodies becoming positive were observed, but our follow-up was only 1 year. Valcavi et al.^(^ ^11^ ^)^ observed a newly developed TgAb and TPOAb of 8.2% and 5.9%, respectively, after 3 years. Longer follow-up will be needed for this purpose.

Our side effects were limited and most related to pain. Our protocol included subcapsular anesthesia with 2% lidocaine. It has been recently demonstrated by a large series of patients that the use of local pericapsular anesthetic increases threefold the risk of pain.^(^ ^14^ ^)^ This requires further investigation with controlled study. We had one patient with burn on the site of the needle insertion, witch was attributed to a technique problem, probably the laser tip was too close to the needle tip. Our study had some limitations, such as a short follow-up period. Another limitation was the lack of a control group (surgery or clinical and ultrasound observation) to compare the results.

## CONCLUSION

Percutaneous laser ablation is safe and effective in the treatment of benign thyroid nodules with improvement in compressive and aesthetic symptoms. Single session percutaneous laser ablation is a promising outpatient minimally invasive treatment of benign thyroid nodule.
